# Editorial: Women in oral health promotion

**DOI:** 10.3389/froh.2024.1454579

**Published:** 2024-07-16

**Authors:** Moréniké Oluwátóyìn Foláyan, Joana Cunha-Cruz

**Affiliations:** ^1^ Department of Child Dental Health, Obafemi Awolowo University, Ile-Ife, Nigeria; ^2^ Department of Clinical & Community Sciences, School of Dentistry, University of Alabama, Birmingham, AL, United States

**Keywords:** women in science, research productivity, sustainable development goals, gender equality, refugee healthcare

**Editorial on the Research Topic**
Women in oral health promotion

Research productivity serves as a crucial indicator of intellectual wealth and economic advancement, directly impacting the health and well-being of a nation's populace. Traditionally, men have tended to publish more frequently and receive higher citation counts than women (1). At present, less than 30% of researchers worldwide are women (2). Long-standing biases and gender stereotypes continue to discourage females from entering science-related fields (3). Yet, as highlighted by the United Nations Educational, Scientific and Cultural Organization, gender equality in science is important for the sustainable development of our planet (4). Promoting gender equality, defeating stereotypes, and ensuring equal representation of women in research are some of the steps in changing traditional mindsets. In this context, Frontiers in Oral Health offered this platform to promote the work of women researchers across all fields of Oral Health Promotion.

The research presented in this special collection showcases the diversity and depth of studies conducted by women in Oral Health Promotion. This research topic received four articles authored by 15 researchers of which seven (46.7%) were women. These women work in Saudi Arabia, Nigeria, Scotland, Egypt, and China. The studies authored by them not only advance theoretical knowledge and experimental methodologies but also address compelling problems in global health. One of the publications by Folayan et al. titled “Gender Differences in Dentistry and Oral Sciences Research Productivity by Researchers in Nigeria” strongly aligns with the focus of our research team. Their bibliometric analysis revealed that the average number of publications and citations per female author in dentistry and oral sciences indexed in the Web of Science was higher than that of male authors. Interestingly, a greater percentage of females were identified as first authors, whereas males predominated as last authors. The authors postulated that gender-related cultural and economic context may explain their observation, which needs further exploration.

Yuan et al.'s study, on the other hand, applied the Transactional Model of Stress and Coping to understand the relationship between dental anxiety and COVID-19 anxiety, using data from dental patients in East China. Their study confirmed the reliability and validity of the COVID-19 Anxiety Scale and the newly developed Clinical care COVID-19 Anxiety Scale showing that dental and COVID-19 anxieties are interrelated, with higher anxiety levels in women and those with lower education. These study findings reinforce the need for healthcare providers to understand anxiety factors to improve patient care during pandemics through measures such as infection control, effective communication, and alternative service delivery methods like tele-dentistry.

Asfari et al. explored refugees' experience of accessing dental health services in host countries. This is a topical issue concerning healthcare disparities among displaced populations. It addresses several sustainable developmental goals (SDG). SDG goal 3 aims to ensure healthy lives and promote well-being for all at all ages. Access to dental health services contributes to overall health and quality of life for refugees. It addresses SDG 10 that states that providing equitable access to healthcare, including dental services, helps reduce inequalities within and among countries, particularly among displaced populations. The topic also addresses the SDG 16 that focuses on ensuring that refugees have access to essential services, including healthcare, promotes peaceful and inclusive societies and strengthens institutions that support displaced populations. Asfari's study highlighted the main barriers that significantly limits refugees’ access oral health care, and factors that helped improve access. Like prior studies had highlighted, more studies are needed to understand and address gaps in dental health service access for not only refugees, but also migrants and internally displaced persons (5, 6).

To achieve this however, oral health needs to be prioritised as a fundamental part of general health and a human right (7, 8). This poor prioritisation had led to poor attention being paid to oral health in global and national health policies (9). Gaffar et al. mapped oral health policies for young children in the member countries of the World Dental Federation and showed that only a fraction of the countries had national oral health policies or include oral health in general health policies. Though many countries have programs targeting disadvantaged populations, there was considerable variation in the types and comprehensiveness of services provided. The study underscored the need for better integration of oral health into general health policies and universal health coverage to improve access and reduce disparities.

This collection of articles stimulates new thinking and opens space for more studies. These include the findings by Folayan et al. challenging traditional norms and highlighting the impactful contributions of women in academia; Yuan et al.'s research that underscores the importance of understanding psychological factors in patient care, particularly during pandemics, to enhance healthcare delivery; Asfari et al. research aiming to ensure healthy lives, reduce inequalities, and promote inclusive societies by improving access to essential healthcare services for refugees; and Gaffar et al.'s study that underscored the need for better integration of oral health into broader health policies to achieve universal health coverage and reduce disparities in healthcare access. A common thread cutting through these studies is their advocacy for equality, equitable healthcare access and policy reform, crucial for achieving global health goals. We postulate that women's participation in oral health promotion research may increase the likelihood of conducting studies that promote equity, which is important for advancing healthcare outcomes, and fostering inclusive scientific communities that benefit society. We look forward to studies to confirm or refute this postulation.

Finally, promoting gender equality in oral health promotion research is not just about fairness; it is essential for achieving the sustainable development goals that benefit all of humanity (4). We therefore encourage early, middle and senior career female researchers to continue to enrich the oral health promotion publication landscape, as their contributions are invaluable.

## References

[1] Brück O. A bibliometric analysis of the gender gap in the authorship of leading medical journals. Commun Med (Lond). (2023) 3(1):179. 10.1038/s43856-023-00417-3 38082119 PMC10713825

[2] UNESCO. Women in Science. (2019). Available online at: https://uis.unesco.org/en/topic/women-science (Accessed June 14, 2024).

[3] Charlesworth TES Banaji MR. Gender in science, technology, engineering, and mathematics: issues, causes, solutions. J Neurosci. (2019) 39(37):7228–43. 10.1523/JNEUROSCI.0475-18.2019 31371423 PMC6759027

[4] OECD Library. Gender equality and sustainable development. In: Gender and the Environment: Building Evidence and Policies to Achieve the SDGs. 21 May 2021. 10.1787/3d32ca39-en

[5] Folayan MO Schroth RJ Ayouni I Nguweneza A Arheiam A Al-Batayneh OB A scoping review linking early childhood caries to violence, neglect, internally displaced, migrant and refugee status. BMC Oral Health. (2023) 23(1):747. 10.1186/s12903-023-03459-0 37821894 PMC10568772

[6] Keboa MT Hiles N Macdonald ME. The oral health of refugees and asylum seekers: a scoping review. Global Health. (2016) 12(1):59. 10.1186/s12992-016-0200-x 27717391 PMC5055656

[7] World Health Organization. Global Oral Health Status Report: Towards Universal Health Coverage for Oral Health by 2030. Geneva: World Health Organization (2022).

[8] Jean G Kruger E Lok V. Oral health as a human right: support for a rights-based approach to oral health system design. Int Dent J. (2021) 71(5):353–7. 10.1016/j.identj.2020.12.021 33612265 PMC9275327

[9] Benzian H Hobdell M Holmgren C Yee R Monse B Barnard JT Political priority of global oral health: an analysis of reasons for international neglect. Int Dent J. (2011) 61(3):124–30. 10.1111/j.1875-595X.2011.00028.x 21692782 PMC9374826

